# Kinase inhibitors can produce off-target effects and activate linked pathways by retroactivity

**DOI:** 10.1186/1752-0509-5-156

**Published:** 2011-10-04

**Authors:** Michelle L Wynn, Alejandra C Ventura, Jacques A Sepulchre, Héctor J García, Sofia D Merajver

**Affiliations:** 1 Center for Computational Medicine and Bioinformatics, University of Michigan Medical School, Ann Arbor, MI, USA; 2 Laboratorio de Fisiología y Biología Molecular, Departamento de Fisiología, Biología Molecular y Celular, IFIBYNE-CONICET, Facultad de Ciencias Exactas y Naturales, Universidad de Buenos Aires, Ciudad Universitaria, Pabellón 2, Buenos Aires, Argentina; 3 Institut Non Linéaire de Nice, Université de Nice Sophia-Antipolis, UMR CNRS 6618, Valbonne, France; 4 Electrical Engineering and Computer Science, University of Michigan, Ann Arbor, MI, USA; 5 Department of Internal Medicine, Division of Hematology and Oncology and Comprehensive Cancer Center, University of Michigan Medical School, Ann Arbor, MI, USA

## Abstract

**Background:**

It has been shown in experimental and theoretical work that covalently modified signaling cascades naturally exhibit bidirectional signal propagation via a phenomenon known as retroactivity. An important consequence of retroactivity, which arises due to enzyme sequestration in covalently modified signaling cascades, is that a downstream perturbation can produce a response in a component upstream of the perturbation without the need for explicit feedback connections. Retroactivity may, therefore, play an important role in the cellular response to a targeted therapy. Kinase inhibitors are a class of targeted therapies designed to interfere with a specific kinase molecule in a dysregulated signaling pathway. While extremely promising as anti-cancer agents, kinase inhibitors may produce undesirable off-target effects by non-specific interactions or pathway cross-talk. We hypothesize that targeted therapies such as kinase inhibitors can produce off-target effects as a consequence of retroactivity alone.

**Results:**

We used a computational model and a series of simple signaling motifs to test the hypothesis. Our results indicate that within physiologically and therapeutically relevant ranges for all parameters, a targeted inhibitor can naturally induce an off-target effect via retroactivity. The kinetics governing covalent modification cycles in a signaling network were more important for propagating an upstream off-target effect in our models than the kinetics governing the targeted therapy itself. Our results also reveal the surprising and crucial result that kinase inhibitors have the capacity to turn "on" an otherwise "off" parallel cascade when two cascades share an upstream activator.

**Conclusions:**

A proper and detailed characterization of a pathway's structure is important for identifying the optimal protein to target as well as what concentration of the targeted therapy is required to modulate the pathway in a safe and effective manner. We believe our results support the position that such characterizations should consider retroactivity as a robust potential source of off-target effects induced by kinase inhibitors and other targeted therapies.

## Background

Cells propagate information through protein signaling pathways that are part of complex signal transduction networks [1]. The simplest view of cellular signaling entails a cascade of molecular events initiated by the recognition of a stimulus and culminating in the chemical alteration of an effector molecule. In the case of covalent modification by the addition or removal of a phosphate group, a reaction commonly found in signaling cascades, each phosphorylated protein serves as the kinase that activates the next cycle's unphosphorylated protein.

Targeted therapies are used to modulate disease progression by inhibiting a specific protein within a dysregulated signaling pathway [2]. Kinase inhibitors are a class of targeted therapies designed to interfere with a specific kinase molecule. While extremely promising as anti-cancer agents, kinase inhibitors can produce off-target effects by inducing changes in molecules other than the one specifically targeted. Such off-target effects are generally attributed to non-specific binding or to cross-talk [3].

**Figure 1:**
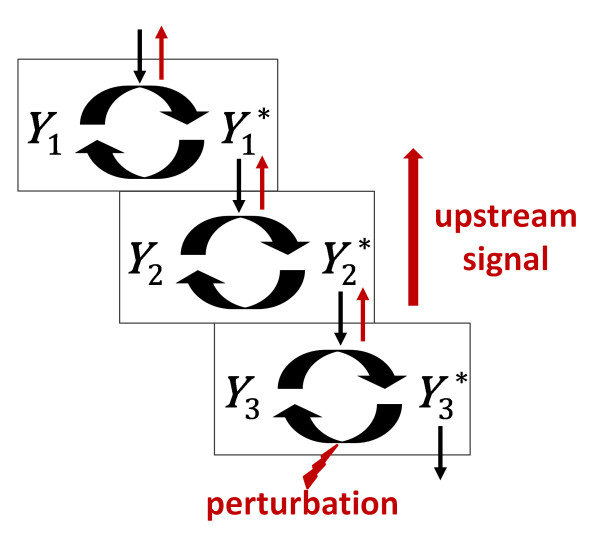
**Retroactivity arises due to enzyme sequestration in covalently modified cascades**. A simple signaling cascade is depicted where each sequential cycle represents the activation (denoted by *) and inactivation of protein *Y_i_*. *Y*_1_* serves as the activating enzyme of *Y*_2 _and *Y*_2_* serves as the activating enzyme of *Y*_3_. The cycles can be thought of as modules where each module's substrate sequesters a key component of the previous module, limiting the component's ability to participate in the previous module. This sequestration induces a natural change in the preceding module which may propagate upstream through one or more preceding modules. In this example, a perturbation in the deactivation reaction of cycle 3 induces an effect in cycle 2. If the perturbation takes the form of an increase in the concentration or activity of the enzyme catalyzing the conversion of *Y*_3_* to *Y*_3_, more *Y*_3 _will be available to react with and sequester *Y*_2_*, resulting in less *Y*_2 _substrate availability for the reaction with *Y*_1_*. Thus, a reverse response can propagate upstream to a preceding cycle or cycles. In the schematic, black arrows represent the cell surface to nucleus direction of cellular signaling and red arrows represent the direction of retroactive signaling.

Recent theoretical and experimental studies have demonstrated that covalently modified cascades also exhibit bidirectional signal propagation via a phenomenon termed retroactivity [4-9]. This phenomenon arises because cycles in a cascade are coupled, not only to the next cycle, but also to the previous cycle (Figure 1). The cycles can be thought of as modules where each module's substrate sequesters a key component of the previous module, limiting the component's ability to participate in the previous module and inducing a natural change in the preceding module. This change may then propagate upstream through one or more preceding modules.

While retroactivity is naturally present in covalently modified cascades, signaling pathways likely have evolved to propagate signals in an optimized downstream manner. An important consequence of retroactivity, however, is that a downstream perturbation in a signaling cascade can produce an upstream effect without the need for explicit negative feedback connections [4]. Retroactivity may, therefore, play important roles in the dysregulated signaling networks of diseased cells as well as the cellular response to targeted therapies applied to dysregulated signaling networks.

Ventura, Sepulchre, and Merajver [4] demonstrated that increasing the concentration of the inactivating enzyme (e.g., a phosphatase) in the terminal cycle of a cascade can decrease the concentration of the activated protein in the previous cycle [4]. This finding led us to hypothesize that a targeted inhibitor can produce upstream off-target effects via retroactivity that can propagate elsewhere in the signaling network.

Off-target effects associated with targeted therapies are often attributed to crosstalk, which refers to inter-pathway molecular interactions arising because of explicit regulatory feedback connections between two pathways or because two pathways share one or more molecular components. It is well accepted that two pathways sharing one or more components can exhibit cross-talk with respect to a stimulation or perturbation above the shared component(s). If an upstream perturbation occurs in one of the pathways, the perturbation may affect the other pathway via the shared downstream component(s). Such a scenario could lead to specificity problems [10]. Here we propose that perturbations (e.g., from an inhibitor) that occur downstream of a shared component can also induce cross-talk effects without any explicit feedback connections via the following mechanism: the information travels upstream from the site of the perturbation through retroactivity, reaches the common component and then is delivered to the parallel pathway.

To test our hypothesis, we created a computational model that tested the application of a kinase inhibitor in a series of simple signaling networks. The objective of the model was to probe the effect of a targeted inhibitor on retroactive signaling and to test whether retroactivity is likely to contribute to measurable off-target effects under physiological conditions. Specifically, the model simulated the targeted inhibition of a specific kinase in a series of multi-cycle networks. In all networks, at least two cascades were activated by the same upstream cycle with no explicit feedback connections between them. Our results indicate that within physiologically and therapeutically relevant ranges for all parameters, a targeted inhibitor can naturally induce a steady state off-target effect via retroactivity. Our results also reveal the surprising and crucial result that a downstream kinase inhibitor has the capacity to turn "on" an otherwise "off" parallel cascade when two cascades share an upstream activator.

## Methods

### Model development

We designed simple signaling networks to test whether a measurable off-target effect in one cascade can occur when a protein in another cascade is selectively inhibited. In each network studied, cycle *i *contained the active (phosphorylated) and inactive (unphosphorylated) forms of protein *Y_i_*, where the active form was denoted by $Y_{i}^{*}$. For simplicity, we refer to activating and inactivating enzymes in a network as kinases and phosphatases, respectively.

**Figure 2:**
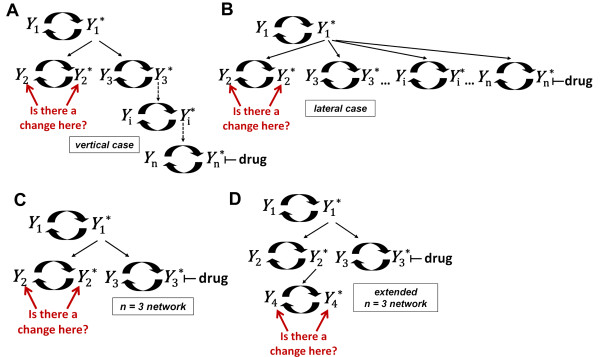
**Topology of signaling networks studied**. Two general types of network motifs consisting of covalently modified cycles were studied: **(A) **the vertical case where the *n*-th cycle in the right hand cascade is inhibited and **(B) **the lateral case where the *n*-th single-cycle cascade is inhibited. **(C) **The *n *= 3 network consisted of exactly 3 cycles and was the simplest form of both the vertical and lateral case. **(D) **An *extended n *= 3 network was also studied where a fourth cycle activated by *Y*_2_* was added to the left-most cascade. In all networks, *Y*_1_* served as the upstream activator and cycle 2 and cycle 3 were always in distinct cascades. No additional regulatory connections were included in any network. Off-target effects in cycle 2 were monitored by measuring the steady state concentrations of *Y*_2 _and *Y*_2_* as the concentration of an inhibitory drug that specifically targeted Y_n_* was increased.

Protein *Y*_1_* served as the activating kinase for all cascades. Cycle 2 and cycle *n *were always in distinct cascades (Figure 2). To determine if an off-target effect occurred due to perturbation by the inhibitor, the steady state concentration of the protein in cycle 2 was monitored as the concentration of the drug that specifically targeted Y_n_* was increased. A competitive inhibitor was used that directly bound to Y_n_*, limiting its ability to participate in the phosphatase reaction of cycle *n*, but did not change the rate of the phosphatase reaction in cycle *n*.

Two general network types were considered: a vertical and a lateral case (Figure 2). The vertical case consisted of two cascades where the inhibited cascade length varied (Figure 2A). This motif is similar to the upstream activation of JUN and P53 by JNK1/2 in the mitogen-activated protein kinase (MAPK) pathway [11]. The lateral case was equivalent to a fan-out network topology and consisted of *n *- 1 single cycle cascades that were all activated by *Y*_1_* (Figure 2B). This motif is similar to the activation of multiple cascades by p38 [11]. The *n *= 3 network consisted of exactly 3 cycles and represented the simplest form of both network types studied (Figure 2C).

The general reaction scheme used for the vertical, lateral, and *n = 3 *networks was:

$$\begin{array}{c} Y_{i}+E_{k_{i}}\underset{d_{i}}{\overset{a_{i}}{↔}}C_{i}\overset{k_{i}}{\underset{}{\to }}Y_{i}^{*}+E_{k_{i}}\\ Y_{i}^{*}+E_{p_{i}}\underset{{d^{\prime \prime }}_{i}}{\overset{{a^{\prime }}_{i}}{↔}}{C^{\prime }}_{i}\overset{k_{i}}{\underset{}{\to }}Y_{i}+E_{p_{i}}\\ Y_{n}^{*}+D\underset{k_{off}}{\overset{k_{on}}{↔}}C_{D} \end{array}$$

Where

$$E_{k_{i}}=\left\{\begin{array}{c} i=1,\\ i=2,i=3,\\ i>3\;\left(\text{vertical}\right),\\ i>3\;\left(\text{lateral}\right), \end{array}\;\begin{array}{c} E_{k_{1}}\\ Y_{1}^{*}\\ Y_{i-1}^{*}\\ Y_{1}^{*} \end{array}\right)$$

$Y_{i}^{*}$ is the activated protein in the *i*^th ^cycle

*Y_i _*is the inactivated protein in the *i*^th ^cycle

$E_{k_{i}}$ is the kinase enzyme in the *i*^th ^cycle

$E_{p_{i}}$ is the phosphatase enzyme in the *i*^th ^cycle

*D *is the inhibitory drug

*C_i _*is the *Y_i _*and $E_{k_{i}}$complex in the *i*^th ^cycle

$C_{i}^{\prime }$ is the $Y_{i}^{*}$ and $E_{p_{i}}$ complex in the *i*^th ^cycle

*C_D _*is the $Y_{n}^{*}$ and *D *complex in the *n*^th ^cycle

### Parameter definitions

In order to reduce the complexity of each network studied, parameters were non-dimensionalized into 4 parameter types as described in Appendix A. The allowed value of each parameter type was restricted to the default ranges listed in Table 1. A summary of the parameter types is provided below.

**Table 1:** The parameter space of each network consisted of a set of non-dimensional parameters, each with a minimum and maximum allowed value.

	*default range*		
		
*parameter*	minimum	maximum	*description*
***E_i_***	0.01	100	total kinase to total substrate ratio
***E'_i_***	0.01	100	total phosphatase to total substrate ratio
***K_i_***	0.01	100	normalized K_m _of kinase reaction
***K'_i_***	0.01	100	normalized K_m _of phosphatase reaction
***P_i_***	0.1	10	ratio of the kinase reaction V_max _to the phosphatase reaction V_max_
***K_B_***	0.01	100	normalized drug disassociation constant

Each cycle *i *consisted of 5 dimensionless parameters: ***E_i_***, ***E'_i_***, ***K_i_***, ***K'_i_***, and ***P_i_***. A final parameter, ***K_B_***, applied to the targeted inhibitor. Randomly selected dimensionless parameter values could not exceed the default ranges listed for each parameter type.

Subscripts containing *k *or *p *indicate parameters associated with a kinase or phosphatase reaction, respectively, and subscripts containing *T *indicate the total concentration of a species. V_max _and K_m _are the standard Michaelis-Menten constants representing, respectively, the maximum velocity of a reaction (at a given enzyme concentration) and the substrate concentration necessary to achieve $\frac{1}{2}V_{\max}$[12].

(1) total enzyme to substrate ratio of the kinase and phosphatase reaction, respectively, in cycle *i*:

$$E_{i}=E_{k_{iT}}∕Y_{iT}\;E_{i}^{\prime }=E_{p_{iT}}∕Y_{iT}$$

(2) normalized K_m _of the kinase and phosphataste reaction, respectively, in cycle *i*:

$$K_{i}=K_{m_{k_{i}}}∕Y_{iT}\;K_{i}^{\prime }=K_{m_{p_{i}}}∕Y_{iT}$$

where $K_{m_{k_{i}}}=\frac{d_{i}+k_{i}}{a_{i}}$ and $K_{m_{p_{i}}}=\frac{{d^{\prime }}_{i}+{k^{\prime }}_{i}}{{a^{\prime }}_{i}}$

(3) V_max _ratio of the kinase and phosphatase reactions in cycle *i*:

$$P_{i}=V_{\max_{k_{i}}}∕V_{\max_{p_{i}}}$$

where $V_{\max_{k_{i}}}=k_{i}E_{k_{iT}}$ and $V_{\max_{p_{i}}}=k_{i}^{\prime }E_{p_{iT}}$

(4) normalized disassociation constant of the inhibitor binding to Y_n_*:

$$K_{B}=\frac{k_{off}∕k_{on}}{Y_{nT}}$$

***E_i _***and ***E'_i _***values less than 1 indicate that the enzyme is less abundant than the substrate. ***K_i _***and ***K'_i _***values less than 1 indicate that the total available substrate exceeds the concentration needed to reach K_m _and, consequently, the enzymatic reaction operates close to or in the zero order regime [13]. In contrast, ***K_i _***and ***K'_i _***values greater than 1 indicate that an insufficient amount of substrate exists to reach K_m _and the enzymatic reaction operates in the linear regime [13]. ***P_i _***values greater than 1 indicate that the V_max _of the kinase reaction exceeds the V_max _of the phosphatase reaction and, consequently, the cycle tends toward the activation reaction. Likewise, ***P_i _***values less than 1 indicate that the cycle tends toward the deactivation reaction.

### Determination of off-target effects

The concentrations of species *Y_i_*, $Y_{i}^{*}$, and the inhibitory drug *D *were normalized as follows:

$$y_{i}=\frac{\left[Y_{i}\right]}{Y_{iT}}\;y_{i}^{*}=\frac{\left[Y_{i}^{*}\right]}{Y_{iT}}\;I=\frac{D_{T}}{Y_{nT}}$$

To determine if a detectable off-target effect occurred for a specific set of parameters, changes in the steady values of *y*_2 _and $y_{2}^{*}$ were monitored as the model parameters were held fixed but ***I ***was varied from 10^-4 ^to 10^4^. If a change in the steady state value of *y*_2 _or $y_{2}^{*}$ occurred that was greater than or equal to a detection threshold of 0.10 (i.e., 10% of the total protein in cycle 2), an off-target effect in cycle 2 was reported. For numeric reasons, the range used for ***I ***was intentionally larger than needed. For a given parameter set, it was numerically more efficient to simulate with a small (10^-4^) and a large (10^4^) value for ***I ***and then check for a change in the steady state values of *y*_2 _and $y_{2}^{*}$ than it was to simulate with many values of ***I***. In fact, the majority of off-target effects in our model were observed as ***I ***was varied from 0.1 to 10.

When we tested the *n = 3 *network, we obtained the same results when we used either ***I ***= 0.0000 or ***I ***= 0.0001 (10^-4^) as the minimum drug concentration. For this reason (and because it would be experimentally challenging to distinguish 0.0000 from 0.0001 *in vivo*), we effectively considered ***I ***= 10^-4 ^to represent the absence of the drug in the system.

### Numerical simulations

For each network tested, a system of ordinary differential equations (ODEs) was used to model the rate of change of the reactants. Because we were only interested in changes in steady state values as a function of ***I***, we first solved the system by setting the ODEs equal to zero and generating a system of steady state equations. As described in Appendix A, the model in this form was the basis for the non-dimensionalization of model parameters.

For numerical reasons, it was more efficient to solve the ODEs over a very long time period rather than solving the system of steady state equations directly. After randomly selecting a set of non-dimensional parameters, the selected values were mapped to corresponding dimensional parameter values (Additional File 1) and the system of ODEs was solved using the Matlab R2009b ode15s stiff solver from 0 to a maximum of 100,000 arbitrary time units. The majority (~90%) of randomly selected parameter sets obtained steady state within 5,000 arbitrary time units. The units are arbitrary because we began with dimensionless parameters lacking an explicit timescale. Finally, to confirm the numerical steady state solution, the original dimensionless parameters and the final *y_i _*and y_i_* variable values were substituted into the analytical steady state equations listed in Appendix A. Matlab source code was compiled as a C program and run on Intel Nehalem/i7 Core processors.

### Random parameter space exploration

Random parameter selection was performed via latin hypercube sampling (LHS) to provide an efficient and even sampling distribution across the range of allowed values in the parameter space [14-16]. Each parameter space exploration consisted of 5000 randomly selected parameter sets. The number of parameter sets sampled was determined by calculating the percent of off-target effects in *q *randomly sampled parameter sets for the *n = *3 network (Figure 2C). The variation in the percent of off-target effects stabilized when *q *was greater than or equal to 5000 (Additional File 2 Figure S1). The percentage of 5000 randomly selected parameter sets that produced an off-target effect provided a probability that off-target effects would occur in a given network's parameter space.

### Numeric perturbation analysis

A modified perturbation method was used to probe which model parameters were most important for producing an off-target effect as a result of the inhibition of Y_n_*. Traditional biochemical sensitivity analysis [17] with the dimensionless parameters was not possible because these parameters applied to the steady state equations and not the time dependent differential equations (Appendix A). Instead, we developed a numerical perturbation based method that allowed us to evaluate the parametric sensitivity of off-target effects in a network's parameter space. In the method, the value of a single parameter was randomly selected from a restricted range of values while all other parameter values were randomly selected from the full range permitted by the baseline parameter space. If off-target effects are sensitive to a given parameter, we expect that when values for the parameter under test are randomly selected from a reduced range of values, the percentage of off-target effects produced will differ from the percentage produced when values for the parameter are instead selected from a fixed baseline range. In both cases, all other parameter values are selected from a fixed baseline range so that the only change in the system is a perturbation in the allowed range of the parameter under test.

The reduced ranges used to perturb each parameter were arrived at by partitioning the default range established for each parameter type in Table 1. The default ranges were divided into smaller perturbation sub-ranges such that the minimum and maximum of a sub-range was an order of magnitude larger than the minimum and maximum of the previous sub-range. Because the ***E_i_***, ***E'_i_***, ***K_i_***, ***K'_i_***, and ***K_B _***parameters had a default initial range of 0.01 - 100.0 (Table 1), the sub-ranges used to perturb these parameters were: (i) 0.01 - 0.10, (ii) 0.10 - 1.0, (iii) 1.0 - 10.0, and (iv) 10.0 - 100.0. Because the ***P_i _***parameters had a default range of 0.10 - 10.0, the sub-ranges used to perturb these parameters were: (i) 0.10 - 1.0 and (ii) 1.0 - 10.0.

A complete numeric perturbation analysis of a parameter space consisted of determining the percentage of off-target effects in 5000 randomly selected parameter sets for each parameter's sub-ranges. In the *n *= 3 network (Figure 2C) there were a total of 16 parameters (5 parameters per cycle and ***K_B_***). Three of the parameters (***P*_1_**, ***P*_2_**, and ***P*_3_**) had 2 perturbation sub-ranges each and the remaining 13 parameters had 4 perturbation sub-ranges each. In this example, the analysis consisted of a total of 59 sets of 5000 simulations (58 sets for each parameter sub-range and 1 set to establish the baseline percentage of off-target effects in the parameter space prior to perturbation).

## Results

The question we wanted to answer with our models was whether a targeted inhibitor is likely to induce a measurable off-target effect due to retroactivity in a non-targeted cascade under physiological conditions. In each network, cycle *n*, was perturbed by an inhibitor. An off-target effect occurred in the model if, after increasing ***I ***(the normalized inhibitor concentration) from 10^-4 ^to 10^4^, a change in the steady state concentration of *Y*_2 _and/or *Y*_2_* occurred that was at least 0.10 of the total *Y*_2 _protein pool. For example, a change of 0.25 in *Y*_2 _and 0.08 in *Y*_2_* would indicate that the steady state values of *Y*_2 _and *Y*_2_* changed by 25% and 8% of the total *Y*_2 _protein pool, respectively, and that a detectable off-target effect occurred in *Y*_2_.

### Specific parameter ranges promote off-target effects in cycle 2

**Figure 3:**
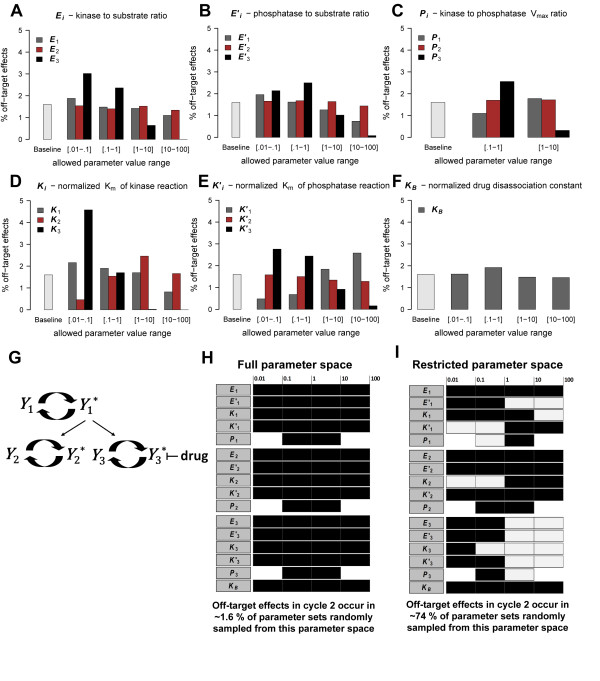
**A numeric perturbation analysis revealed parameter value ranges that promote off-target effects in the *n *= 3 network**. A perturbation analysis of the *n = 3 *network **(G) **was performed where a single parameter's value was randomly selected from a small range of values, while all other parameters were selected from the larger ranges defined in Table 1. The baseline in each plot reflects the percent of off-target effects in cycle 2 in 5000 sampled parameter sets when all parameter values were randomly selected from the ranges defined in Table 1 and depicted in **(H)**. All other bars reflect the results of systematically perturbing each parameter (one at a time) using the given sub-ranges **(A-F)**. Based on this perturbation analysis, a restricted parameter space was generated **(I) **from which ~74% of the sampled parameter sets produced off-target effects in cycle 2. In contrast, only ~1.6% of sampled parameter sets from the full parameter space **(H) **produced off-target effects in cycle 2.

First, we investigated the *n *= 3 network (Figure 2C) where *Y*_3_* is targeted by the inhibitor. When the full parameter space (defined in Table 1 and depicted in Figure 3H) was used, 1.6% of the 5000 randomly selected parameters sets produced an off-target effect in cycle 2. This value was essentially unchanged (1.5%) when we randomly selected 50,000 parameter sets for comparison (Additional File 2). To identify the model parameters that were most important for producing a cycle 2 off-target effect, a numeric perturbation analysis was performed (Figure 3A-F). The results of the analysis suggest that the parameters controlling the activity of cycle 3 play a large role in inducing an off-target effect in cycle 2. Not surprisingly, ***K*_3 _**(the normalized K_m _of the kinase reaction in cycle 3) had the greatest effect on off-target effects in this network (Figure 3D). ***K*_3 _**determines how much sequestration of *Y*_1_* by *Y*_3 _occurs and this is the key mechanism of retroactivity. When ***K*_3 _**was restricted to values greater than 1, the off-target effects in the network were essentially eliminated. In contrast, when ***K*_3 _**was restricted to values between 0.01 and 0.10, the percentage of off-target effects increased to 4.6%. Similarly, ***K'*_3 _**(the normalized K_m _of the phosphatase reaction in cycle 3) also affected the percentage of off-target effects but to a lesser degree than ***K*_3 _**(Figure 3E).

***E*_3 _**and ***E'*_3 _**(the total kinase to substrate and the total phosphatase to substrate ratio, respectively, in cycle 3) also appeared to exert a large degree of control over off-target effects (Figure 3A-B). These results indicate that off-target effects were more likely when the kinase and phosphatase enzymes of cycle 3 were saturated. ***P*_3_**, the ratio of the V_max _of the kinase and phosphatase reactions in cycle 3, also affected the percentage of off-target effects (Figure 3C). When ***P*_3 _**was less than 1, cycle 3 tended toward the deactivation reaction and the percentage of off-target effects increased to 2.56% from 1.6%. Similarly, when ***P*_3 _**was greater than 1, cycle 3 tended toward the activation reaction and the percentage of off-target effects was significantly reduced relative to the baseline (0.32%).

The only parameter associated with cycle 2 that affected the percentage of off-target effects in this network was ***K*_2 _**(the normalized K_m _of the kinase reaction of cycle 2). ***K*_2 _**values between 0.01 and 0.10 are expected to produce an efficient kinase reaction because $Y_{2T}>>K_{m_{2}}$. The results of the numerical perturbation analysis indicated that when ***K*_2 _**was restricted to values in this sub-range, a small percentage of off-target effects was observed (Figure 3D). In contrast, when ***K*_2 _**was restricted to values between 1.0 and 10.00, the percentage of off-target effects increased relative to the baseline. These results suggest that an off-target effect in cycle 2 is more likely to occur in the *n *= 3 network when the conversion of *Y*_2 _to *Y*_2_* operates in the linear regime because of substrate constraints. This result is somewhat counter intuitive given the fact that we are interested in measuring a response that propagates from cycle 3 to cycle 1 and then down to cycle 2. It is reasonable to expect that an efficient kinase reaction in cycle 2 would be important for recruiting *Y*_1_* to activate *Y*_2 _and generate an effect in cycle 2. If the cycle 2 kinase reaction is less efficient than the cycle 3 kinase reaction, however, more *Y*_1_* will be available to convert *Y*_3 _to *Y*_3_*, ultimately contributing to the sequestration of more *Y*_3_* into a complex with *D*. Such a sequestration may give rise to a detectable upstream effect as a result of the reduced substrate availability in cycle 3.

The cycle 1 parameters with the greatest impact on the percentage of off-target effects were ***K*_1 _**and ***K'*_1 _**(the normalized K_m _of the kinase and phosphatase reaction, respectively, in cycle 1) (Figure 3D-E). Larger values of ***K*_1 _**acted to suppress off-target effects, while smaller values produced an increase in off-target effects relative to the baseline (Figure 3D). The reverse was observed for ***K'*_1_**, with higher values producing a higher percentage of off-target effects than smaller values (Figure 3E). Together, the ***K*_1 _**and ***K'*_1 _**results suggest that off-target effects are favored when the cycle 1 phosphatase reaction tends toward inefficiency and the cycle 1 kinase reaction tends towards efficiency. This result is not surprising given that the availability of *Y*_1_* is essential for the propagation of a signal from cycle 3 to cycle 2.

The value of ***K_B_***, the normalized drug disassociation constant, had a very slight effect on the percentage of off-target effects. In general, ***K_B _***values greater than 1 produced a slight decrease in the percentage of off-target effects relative to the baseline (Figure 3F). This result suggests that weaker binding (and larger dissociation constants) promoted fewer off-target effects, as would be expected given the decreased sequestration of *Y*_3_* that would occur. The change in the percentage of off-target effects induced by restricting ***K_B _***values was fairly small compared to the change induced when other model parameter values were restricted. This result suggests that the activity and efficiency of component cycles in the network may be more important for propagating an off-target effect than the actual kinetics of a targeted therapy.

The results of the above analysis indicate that certain parameter value ranges are more likely to induce an off-target effect in cycle 2 as the drug concentration is increased. When we restricted the *n *= 3 parameter space by reducing the ranges from which some key parameters were selected (Figure 3I), the percentage of off-target effects in 5000 randomly sampled parameter sets increased from 1.6% to 73.9%.

A second numerical perturbation analysis was performed using this new restricted *n *= 3 parameter space as a baseline. In general, many of the trends observed in the analysis of the original *n *= 3 parameter space (depicted in Figure 3H) were observed in the analysis of the restricted *n *= 3 parameter space (Additional File 3 Figure S3). For example, low ***K*_3 _**values remained important for producing off-target effects in both parameter spaces. The effects of parameters associated with cycle 2, however, were different in the two parameter spaces. When the original parameter space was tested, ***K*_2 _**was the only cycle 2 parameter found to substantially affect the percentage of off-target effects (Figure 3D). In the restricted parameter space, however, some ranges of ***E*_2_**, ***E'*_2_**, and ***K'*_2 _**produced off-target effect percentages that differed substantially from the baseline. For example, ***E*_2 _**values between 10 and 100 produced off-target effects in 92.1% of sampled parameter sets, the largest percentage of off-target effects observed in any of our analyses (Additional File 3 Figure S3A). Because ***E*_2 _**is the total enzyme to substrate ratio of the kinase reaction (*Y*_1T_/*Y*_2T_), this result suggests when more total protein exists in cycle 1 compared to cycle 2, off-target effects in cycle 2 are more likely in this network.

While some parameters associated with cycle 2 were able to effect the percentage of off-target effects, the parameters associated with cycle 3 continued to have the greatest effect on off-target effects in the restricted *n *= 3 parameter space. Only parameters in cycle 3 had the ability to effectively eliminate (or substantially reduce) the percentage of off-target effects within specific reduced ranges. Values between 10 and 100 for ***E*_3_**, ***E'*_3_**, ***K*_3 _**and ***K'*_3 _**produced off-target effect percentages of 0%, 3.24%, 0% and 3.20%, respectively. In addition, ***P*_3 _**values greater than 1 produced off-target effects in 18.24% of sampled parameter sets which, compared to the baseline of 73.9%, represents a large decrease in off-target effects (Additional File 3 Figure S3).

### Varying a single parameter can produce a large change in the size of the off-target effect

**Figure 4:**
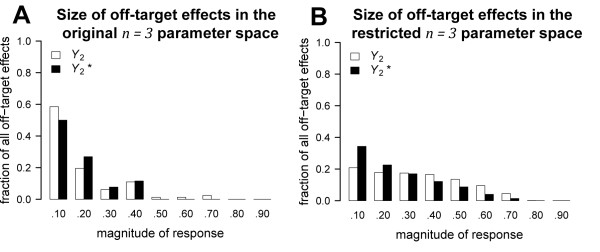
**Distribution of the size of off-target effects in the *n *= 3 network**. Histograms of the size of off-target effects in *n *= 3 network (Figure 3D) are plotted for two different parameter spaces. The y-axis on each plot represents the proportion of all parameter sets that produced off-target effects in 5000 randomly selected parameter sets. The x-axis on each plot represents the size of an off-target effect in cycle 2 as a proportion of *Y*_2T _such that each value indicates a response that was at least as big as the given value but less than the next sequential value. For example, a value of .30 indicates that the magnitude of the response was greater than or equal to .30 but less than 0.40. **(A) **The majority of off-target effects in the original *n *= 3 parameter space (depicted in Figure 3H) were less than 0.30. **(B) **In contrast, the distribution of the size of off-target effects in the restricted *n *= 3 parameter space (depicted in Figure 3I) was more uniform.

The magnitude of off-target effects produced by parameter sets randomly sampled from the original *n *= 3 parameter space (depicted in Figure 3H) generally fell between .10 and .30 of the total *Y*_2 _protein pool (Figure 4A). In contrast, the magnitude of off-target effects produced by parameter sets randomly sampled from the restricted *n *= 3 parameter space (depicted in Figure 3I) were more uniformly distributed across a range of values (Figure 4B). These results suggest that when conditions in a network are favorable for off-target effects, the size of an off-target effect is highly variable.

**Figure 5:**
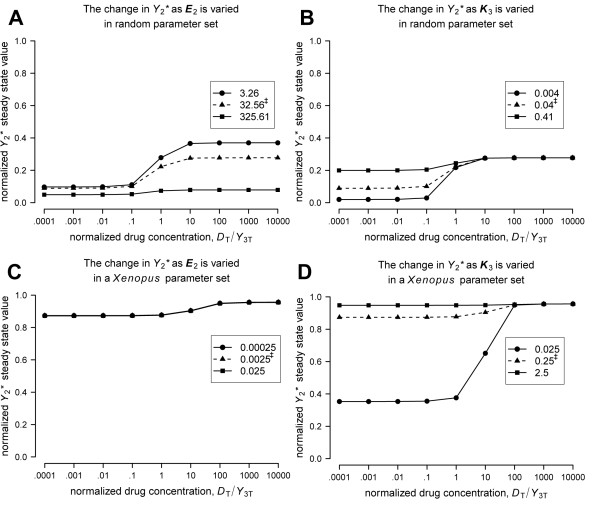
**Varying a single parameter value can produce a large change in the off-target response**. Stimulus response curves were plotted for the *n *= 3 network using a randomly selected parameter set and a parameter set derived from a *Xenopus *model [18] (all parameters values are listed in Table 2). For each parameter set, ***E*_2 _**and ***K*_3 _**were increased or decreased by 1 order of magnitude and the resulting stimulus response curves were plotted. **(A-B) **The random parameter set produced an off-target effect in *Y*_2 _of 0.40 (data not shown) and in *Y*_2_* of 0.19. **(A) **Increasing ***E*_2 _**from 32.56 to 325.61 substantially decreased the off-target effect in *Y*_2_*, while decreasing ***E*_2 _**to 3.26 increased the off-target effect in *Y*_2_* to 0.27. (**B**) Increasing ***K*_3 _**from 0.04 to 0.41 reduced the response in *Y*_2_* below the detection threshold to 0.07, while decreasing ***K*_3 _**to 0.004 increased the off-target response to 0.26. **(C-D) **A second parameter set was derived from the literature using MAPK parameters from a *Xenopus *model. This parameter set did not initially produce an off-target effect because the response in both *Y*_2_* and *Y*_2 _was 0.08, which was below the detection threshold. (**C**) Increasing or decreasing ***E*_3 _**to 0.025 or 0.00025, respectively, from 0.0025 had no effect on the response to the targeted inhibitor. (**D**) In contrast, increasing ***K*_3 _**from 0.25 to 2.5 eliminated the original response completely, while decreasing ***K*_3 _**from 0.25 to 0.025 produced a large off-target response of 0.60. Original parameter values prior to variation are indicated by ^‡ ^on the plots (see also Table 2).

We used stimulus response curves to examine how a change in a single parameter value may affect the size of an off-target effect in *Y*_2_* as a function of the normalized inhibitor concentration (Figure 5). A randomly selected parameter set and a parameter set derived from a *Xenopus *MAPK model [18] were used (refer to Additional File 4 for the derivation of the *Xenopus *parameter values). In each parameter set, either ***E*_2 _**or ***K*_3 _**was varied, while all other parameter values were fixed to the values listed in Table 2.

**Table 2:** Parameter sets used in stimulus response curves.

	**Random set**	***Xenopus *set**
	
***E*_1_**	4.87	0.1
***E*_2_**	**32.56**	**0.0025**
***E*_3_**	0.28	0.0025
***E'*_1_**	0.05	0.1
***E'*_2_**	1.26	0.00025
***E'*_3_**	0.29	0.1
***K*_1_**	5.07	100
***K*_2_**	28.18	0.25
***K*_3_**	**0.04**	**0.25**
***K'*_1_**	66.34	100
***K'*_2_**	9.33	0.25
***K'*_3_**	0.59	0.25
***P*_1_**	0.21	1
***P*_2_**	3.43	1
***P*_3_**	0.42	0.025
***K_B_***	0.05	0.0833

The two parameters sets used in Figure 5 are summarized in the table. The Random set refers to a randomly selected parameter set and the *Xenopus *set refers to a parameter set derived from a *Xenopus *MAPK model (Additional File 4). Bolded values represent the original parameter values varied in Figure 5.

The randomly selected parameter set produced a baseline off-target response of 0.19 in *Y*_2_* (Figure 5A-B) and of 0.40 in *Y*_2 _(data not shown). In this parameter set the original ***E*_2 _**value was 32.56 and the original ***K*_3 _**value was 0.04 (Table 2). Increasing ***E*_2 _**to 326.61 substantially decreased the response in *Y*_2_* and decreasing ***E*_2 _**to 3.26 increased the response in *Y*_2_* from 0.19 to 0.27 (Figure 5A). Similarly, increasing ***K*_3 _**to 0.41 reduced the response in *Y*_2_* to 0.07 (below the detection threshold) and decreasing ***K*_3 _**to 0.004 increased the response in *Y*_2_* to 0.26 (Figure 5B).

The parameter set derived from the MAPK *Xenopus *model [18] produced a baseline response of 0.08 (below the detection threshold) in both *Y*_2_* (Figure 5C-D) and *Y*_2 _(data not shown). In this parameter set the original ***E*****_2 _**value was 0.0025 and the original ***K*_3 _**value was 0.25 (Table 2). While changing ***E*_2 _**did not alter the response (Figure 5C), increasing ***K*_3 _**to 2.5 completely eliminated the response in *Y*_2_* and decreasing ***K*_3 _**to 0.025 substantially increased the response in *Y*_2_* to 0.60 (Figure 5D). These results suggest that when using physiologically realistic parameter values, changing one kinetic parameter or species concentration by an order of magnitude has the capacity to dramatically alter whether a targeted inhibitor induces an off-target effect.

A few of the enzyme to substrate ratios in the *Xenopus *parameter set (***E*_2 _**= 0.0025, ***E'*_2 _**= 0.00025, and ***E*_3 _**= 0.0025) were outside the limits of parameter ranges allowed in our random parameter space explorations (Table 1 and Figure 3H), suggesting that off-target effects are possible for a larger range of parameter values than we specifically tested. While we may have been too conservative in the estimation of the ranges defined in Table 1, this finding supports the position that a targeted inhibitor can naturally induce an off-target effect via retroactivity over a range of physiologically relevant conditions.

### The percentage of off-target effects decreased as the size of the vertical and lateral networks increased

We next investigated networks with more than 3 cycles by randomly exploring the parameter spaces of the vertical (Figure 2A) and lateral (Figure 2B) cases using *n *= 5 and *n *= 7 cycles. As before, we measured the steady state change in cycle 2 as the normalized concentration of the drug that targeted cycle *n *was increased. The restricted parameter space depicted in Figure 3I (from which 73.9% of sampled parameter sets produced off-target effects in cycle 2) was used for this analysis. Networks were analyzed using homogenous parameter values in cycles 4, 5, 6 and 7 that equalled the corresponding parameter values randomly selected for cycle 3 (e.g., in the *n *= 5 case, ***E*_3 _**= ***E*_4 _**= ***E*_5_**). This allowed us to keep the size of the parameter space fixed so that 5000 parameter sets remained a reasonable number to sample from each network's parameter space.

In the vertical case, the percentage of off-target effects in the *n *= 5 and *n *= 7 networks were 27.92% and 13.50%, respectively (Table 3). The reduced probability of off-target effects as the cascade lengthened suggests that applying a targeted inhibitor near the bottom of a long cascade can produce a detectable off-target response but the signal may attenuate as it travels up the cascade. This conclusion is in agreement with a recent work that investigated retroactivity in long signaling cascades [9] and found that retroactive signals are likely to attenuate as they travel up long cascades.

**Table 3:** The percentage of off-target effects decreased as the network size increased.

*n*	*Off Target Effects*	
3	73.9	
	*vertical*	*lateral*
	
5	27.9	6.0
7	13.5	0.0

The *n *= 3 network's restricted parameter space produced 73.9% off-target effects. The *n *= 5 and *n *= 7 vertical case networks produced 27.9% and 13.5% off-target effects, respectively, using the same parameter space. In the lateral case the drop was more dramatic with the *n *= 5 and *n *= 7 networks producing 6% and 0% off-target effects, respectively. Parameter values used in cycles 4, 5, 6 or 7 were homogenous with cycle 3. All percentages are out of 5000 randomly selected parameter sets using the parameter space depicted in Figure 3I.

In the lateral case, the drop in the percentage of off-target effects was more dramatic than in the vertical case, with the *n *= 5 and *n *= 7 networks producing 6% and 0% off-target effects, respectively (Table 3). This result suggests applying a targeted inhibitor to a cycle that is activated by a signaling molecule involved in the simultaneous activation of many other cycles decreases the likelihood of off-target effects. This conclusion is based on a limited exploration of the parameter space (due to the homogenous parameter selection used for cycles 3 and greater) but is in agreement with a model proposed by Kim et al. [8] that showed retroactivity (or what they referred to as subsrate-dependent control) is attenuated by the number of substrates available.

### Off-target effects from retroactivity can propagate down a non-targeted cascade

Our results suggest that, under appropriate conditions, it is possible for a downstream perturbation from a targeted inhibitor to transmit up a cascade resulting in a detectable off-target effect near the top of another cascade. Because signal amplification is an important cellular sensory mechanism [19], we next investigated whether off-target effects from targeted inhibitors are likely to amplify down a non-targeted cascade.

To test for downstream propagation of off-target effects from cycle 2, we created an *extended n *= 3 network by adding a 4^th ^cycle activated by *Y*_2_* (Figure 2D). If a change in the steady state concentration of *Y*_4 _and/or *Y*_4_* occurred that was at least 0.10 of the total *Y*_4 _protein pool, then an off-target effect was considered to have occurred in cycle 4. If an off-target effect occurred in cycle 4 and the size of the response in cycle 4 exceeded the size of the response in cycle 2, then an off-target effect with amplification was considered to have occurred in cycle 4.

When the default parameter ranges defined in Table 1 were used for all cycles in the *extended n *= 3 network, the percentage of off-targets in cycle 2 and cycle 4, respectively, was 1.78% and 0.03%. We next tested the *extended n *= 3 network using the restricted *n *= 3 parameter space (depicted in Figure 3I) for cycles 1 - 3 and the default parameter ranges from Table 1 for cycle 4. In this partially restricted parameter space (depicted in Additional File 3 Figure S4H), the percentage of off-target effects in cycle 2 and cycle 4 were 75.3% and 35.5%, respectively, and amplification contributed to cycle 4 off-target effects in 23.3% of the sampled parameter sets (representing more than half of the off-target effects in the sampled parameter sets). The remaining off-target effects in cycle 4 occurred in 12.2% of the sampled parameter sets and had a response size that was either attenuated relative to cycle 2 or equal to the cycle 2 response (Table 4).

**Table 4:** Off-target effects can amplify downstream of cycle 2.

	*Cycle 2*	*Cycle 4*
Cycles 1-3 with restricted ranges and cycle 4 with default ranges		

*% Off Target Effects*	75.3	35.5
*% Off Target Effects with Amplification*	--	23.4
*% Off Target Effects without Amplification*	--	12.1

Cycles 1- 4 with restricted ranges		

*% Off Target Effects*	45.3	67.4
*% Off Target Effects with Amplification*	--	61.9
*% Off Target Effects without Amplification*	--	5.5

To test for downstream propagation of off-target effects from cycle 2, the *extended n *= 3 network was used. If an off-target effect occurred in cycle 4 and the size of the response in cycle 4 exceeded the size of the response in cycle 2, then an off-target effect with amplification was reported for cycle 4. First, the *n *= 3 restricted parameter space was used for cycles 1 - 3 and the default parameter ranges from Table 1 were used for cycle 4 (Additional File 3 Figure S4H). Next, cycles 2 - 4 were further restricted to ranges which favor off-target effect propagation from cycle 2 to 4 (Additional File 3 Figure S4I). Values listed in the table are percentages out of 5000 randomly selected parameter sets.

To identify the parameters that were most important for amplifying an off-target effect from cycle 2 to cycle 4 in the *extended n *= 3 network, we performed a numeric perturbation analysis (as previously described) on the partially restricted parameter space depicted in Additional File 3 Figure S4H. From these results, we generated a new parameter space (Additional File 3 Figure S4I) which produced off-target effects of 45.3% and 67.4% in cycle 2 and cycle 4, respectively. Amplification contributed to cycle 4 off-target effects in 61.9% of the sampled parameter sets. The remaining off-target effects in cycle 4 occurred in 5.5% of the sampled parameter sets and had a response size that was either attenuated relative to cycle 2 or equal to the cycle 2 response (Table 4).

## Discussion

**Figure 6:**
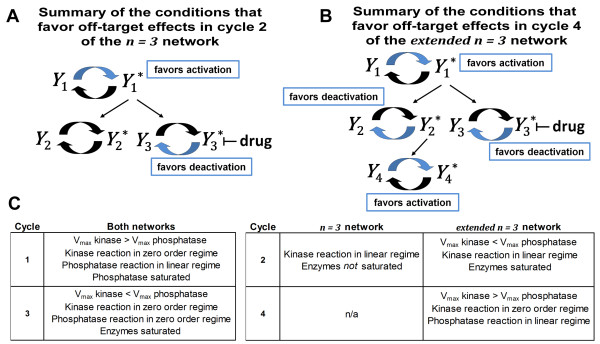
**A summary of conditions that favor off-target effects in the *n = 3 *and the *extended n = 3 *networks**. The conditions that promoted off-target effects in our model are summarized for two network types. Off-target effects in **(A) **the *n *= 3 network and **(B) **the *extended n *= 3 network were favored when cycle 3 tended toward deactivation and cycle 1 tended toward activation. Off-target effects were favored in cycle 4 of **(B) **the *extended n *= 3 network when cycle 2 tended toward deactivation and cycle 4 tended toward activation. Blue arrows in a cycle indicate which V_max _is larger when off-target effects are favored. **(C) **A summary of the specific conditions in each cycle found to favor off-target effects in the *n = 3 *network, in the *extended n = 3 *network, or in both networks is also provided.

We developed a computational model to test whether targeted therapies, such as kinase inhibitors, can produce off-target effects in upstream pathways as a consequence of retroactivity alone. Using a numeric perturbation method, we identified specific conditions (Figure 6) that favored the promotion of steady state off-target effects via retroactivity when a targeted inhibitor was applied to cycle *n *in a series of simple signaling networks (Figure 2).

Our investigation considered only the effect of retroactivity and targeted inhibitors on the individual motifs we studied in the absence of genetic and/or other regulatory relationships. This allowed us to investigate whether such motifs have the capacity to produce off-target effects without regulatory feedback connections. In addition, the present study only considered the steady state response to a targeted therapy. The primary reason we considered only steady state responses was because it provided us with an objective measure that could be used to compare the effect of a targeted inhibitor across many different parameter sets. It is important to note that the dynamics of a retroactive signaling process are likely to induce transient changes in the levels of key signaling molecules. These transient changes, which are not observable at steady state, may lead to important *in vivo *responses.

It is also well known that the dynamics of signal transduction networks can be modulated by important oscillatory behavior, for example, from the P53/MDM2 regulatory feedback loop [20,21]. Because we have not considered transient dynamics, our approach cannot be assumed to apply to all signaling networks. Nevertheless, we expect conditions that favor the induction of off-target effects at steady state to also favor the induction of detectable transient changes associated with the steady state response. In fact, this is what we observed when we plotted the time-course of the cycle 2 proteins with the parameter sets used in Figure 5 (data not shown).

This work has led to very interesting and somewhat surprising results. A major importance of this work is that it did not investigate off-target effects related to a specific therapeutic intervention. There are, however, examples of targeted inhibitors of great clinical interest that are involved in signaling motifs similar to the network motifs we examined. The drug NSC 74859 [22], for example, is a selective inhibitor that targets STAT3. JAK is an upstream activator of both STAT3 and PI3K [23], thus when NSC 74859 inhibits STAT3, JAK could potentially facilitate the propagation off-target effects due to retroactive signaling from STAT3 to PI3K. Moreover, the inhibitor GSK690693 [24] targets AKT and could potentially give rise to a retroactive signal that propagates upstream to a common activator of either the MAPK or STAT3 cascades, generating off-target effects in these pathways.

The binding affinity of the inhibitor for its target did not play a substantial role in the promotion of off-target effects in our model. Instead, the kinetics of the component cycles in the network were more important for increasing the likelihood of off-target effects (Figure 3 and Additional File 3 Figure S3, S4). In general, off-target effects were more likely to occur in the networks studied when the targeted cycle *n *favored the deactivation reaction because the V_max _of the deactivation reaction was larger than the V_max _of the activation reaction and/or both enzymatic reactions in cycle *n *operated in or near the zero order regime. Off-target effects were also more likely when cycle 1 (the source of the shared activator in our models) favored the activation reaction and its kinase reaction operated in or near the zero order regime.

If cycle 2's cascade was extended to include cycle 4 (Figure 2D), which was activated by *Y*_2_*, off-target effects were more likely to propagate to cycle 4 when cycle 2 favored deactivation and cycle 4 favored activation. In cycle 2 this meant that the kinetics of the kinase reaction were generally inefficient (operating in or near the linear regime) and that the V_max _of the deactivation reaction was generally larger than the V_max _of the activation reaction. Thus, off-target effects were promoted when cycle 2 was "off" and not consuming significant amounts of the shared upstream activator, Y_1_*.

The results also indicate that off-target effects were more likely when the total kinase to substrate and the total phosphatase to substrate ratios in the inhibited cycle (***E_n _***and ***E'_n_***, respectively) were less than 1. In the *n = 3 *network, this meant that there was less total protein in cycle 1 than in cycle 3 because ***E*_3 _**< 1 implies *Y*_1T _<*Y*_3T_. The reason for this is that the smaller the *Y*_1T_/*Y*_3T _ratio, the stronger the sequestration of *Y*_1_* will be. The impact of this ratio increases if cycle 3 favors the deactivation reaction such that a large fraction of *Y*_3T _is in the inactive *Y*_3 _form, promoting the binding of *Y*_3 _to *Y*_1_*.

The immediate experimental implications of this result is that, in the absence of kinetic information, the likelihood of off-target effects may potentially be estimated for a network configuration of this type (Figure 2A,C-D) based on the ratio of the concentrations of components in the inhibited cycle and the preceding cycle (using, for example, proteomic or gene expression data). While this ratio would not be an absolute predictor, the presence of this condition would suggest an increased probability of off-target effects.

In agreement with the work of other groups [8,9], we found the probability of off-target effects attenuated when the targeted cycle was near the bottom of a long cascade or when there were many substrates competing for a common upstream activator (Table 3). Our results also suggest that within physiologically realistic parameter ranges, changing a single kinetic parameter or species concentration by 1 order of magnitude has the capacity to dramatically alter whether an off-target effect occurs as a direct result of targeted inhibition. It is also worth noting that, even though we varied the normalized drug concentration over a very large range, in general, the normalized inhibitor concentration needed to change by only 2 orders of magnitude to induce an off-target effect (see, for example, Figure 5).

## Conclusions

Off-target drug effects *in vivo *are typically attributed to cross-talk arising from a feedback connection in a signaling network or to non-specific interactions with other proteins. In this work we have demonstrated that off-target drug effects can also arise naturally from retroactivity in a covalently modified signaling network. This view of signaling challenges the widespread notion that information in signaling cascades only flows from the cell surface to the nucleus and, consequently, this work has far reaching implications for targeted cancer therapies.

A crucial finding of this work is that the kinetics governing the covalently modified cycles in a signaling network are likely to be far more important for propagating an off-target effect due to retroactive signaling than the binding affinity of the drug for the targeted protein, which is a commonly optimized property in drug development. Another particularly paramount finding is that an off-target effect due to retroactive signaling is more likely when the first cycle in a non-inhibited cascade is "off" and essentially inactive. This suggests that, in the motifs we studied, a targeted therapy has the capacity to turn "on" an otherwise "off" tributary cascade.

To emphasize, it is entirely possible for a branch of a signaling network that is "off" to become activated or "on" due to the inhibition of another protein in the network based on retroactivity alone, suggesting an inherent opportunity for negative therapeutic effects. Our findings, therefore, have implications for somatic evolution in cancer and the onset of therapeutic resistance, which has been widely reported for many targeted cancer therapeutics [25], most notably for the targeted inhibition of BCR-ABL by imatinib [26]. Moreover, a single mutation could conceivably give rise to a spontaneous off-target effect without the need for any direct regulatory connections between the targeted protein and the effected protein.

While our approach does not definitively establish that the predicted responses will occur *in vivo*, our results demonstrate that off-target effects are indeed possible in the absence of direct regulatory relationships and suggest that additional (and more specific) experimental and theoretical investigations are warranted. A proper characterization of a pathway's structure is important for identifying the optimal protein to target as well as what concentration of the targeted therapy is required to modulate the pathway in a safe and effective manner. We believe our results strongly support the position that such characterizations should consider retroactivity as a potential source of off-target effects induced by kinase inhibitors and other targeted therapies. This work has also provided an initial roadmap for how to assess the likelihood of off-target effects in a signaling network.

## Authors' contributions

ACV and SDM conceived the study. ACV and JAS created the general model. ACV and MLW designed the experiments and analyzed results. MLW, HJG, and ACV wrote codes for numerical simulations. MLW wrote the manuscript and SDM, ACV, and JAS edited the manuscript. All authors read and approved the final manuscript.

## Appendix A - Non-dimensionalization of the *n *= 3 network

In order to reduce the complexity of the networks studied, model parameters were non-dimensionalized. The following explains the non-dimensionalization of the *n *= 3 network. The dimensionless parameters of the *n = 5 *and *n = 7 *vertical and lateral networks' were obtained in a similar manner.

The ODEs and conservation laws governing the *n = 3 *network (Figure 2C) at steady state are:

$$\begin{array}{llll} \frac{d\left[Y_{1}^{*}\right]}{dt} & =k_{1}\left[C_{1}\right]-{a^{\prime }}_{1}\left[Y_{i}^{*}\right]\left[E_{p_{1}}\right]+{d^{\prime }}_{1}\left[{C^{\prime }}_{1}\right]\; & & \;\\ & \;-a_{2}\left[Y_{2}\right]\left[Y_{1}^{*}\right]+\left(d_{2}+k_{2}\right)\left[C_{2}\right]\; & & \;\\ & \;-a_{3}\left[Y_{3}\right]\left[Y_{1}^{*}\right]+\left(d_{3}+k_{3}\right)\left[C_{3}\right]=0\; & & \;\\ & \; & \end{array}$$

$$\frac{d\left[C_{1}\right]}{dt}=a_{1}\left[Y_{1}\right]\left[E_{k_{1}}\right]-\left(d_{1}+k_{1}\right)\left[C_{1}\right]=0$$

$$\frac{d\left[{C^{\prime }}_{1}\right]}{dt}=a_{1}^{\prime }\left[Y_{i}^{*}\right]\left[E_{p_{1}}\right]-\left(d_{1}^{\prime }+k_{1}^{\prime }\right)\left[C_{1}^{\prime }\right]=0$$

$$\frac{d\left[Y_{2}^{*}\right]}{dt}=-a_{2}^{\prime }\left[Y_{2}^{*}\right]\left[E_{p_{2}}\right]+d_{2}^{\prime }\left[C_{2}^{\prime }\right]+k_{2}\left[C_{2}\right]=0$$

$$\frac{d\left[C_{2}\right]}{dt}=a_{2}\left[Y_{2}\right]\left[Y_{1}^{*}\right]-\left(d_{2}+k_{2}\right)\left[C_{2}\right]=0$$

$$\frac{d\left[{C^{\prime }}_{2}\right]}{dt}=a_{2}^{\prime }\left[Y_{2}^{*}\right]\left[E_{p_{2}}\right]-\left(d_{2}^{\prime }+k_{2}^{\prime }\right)\left[C_{2}^{\prime }\right]=0$$

$$\begin{array}{llll} \frac{d\left[Y_{3}^{*}\right]}{dt} & =-{a^{\prime }}_{3}\left[Y_{3}^{*}\right]\left[E_{p_{3}}\right]+{d^{\prime }}_{3}\left[{C^{\prime }}_{3}\right]+k_{3}\left[C_{3}\right]\; & & \;\\ & \;-k_{on}\left[Y_{3}^{*}\right]\left[D\right]+k_{off}\left[C_{D}\right]=0\; & & \;\\ & \; & \end{array}$$

$$\frac{d\left[C_{3}\right]}{dt}=a_{3}\left[Y_{3}\right]\left[Y_{1}^{*}\right]-\left(d_{3}+k_{3}\right)\left[C_{3}\right]=0$$

$$\frac{d\left[{C^{\prime }}_{3}\right]}{dt}=a_{3}^{\prime }\left[Y_{3}^{*}\right]\left[E_{p_{3}}\right]-\left(d_{3}^{\prime }+k_{3}^{\prime }\right)\left[C_{3}^{\prime }\right]=0$$

$$\frac{d\left[C_{D}\right]}{dt}=k_{on}\left[Y_{3}^{*}\right]\left[D\right]-k_{off}\left[C_{D}\right]=0$$

$$Y_{1T}=\left[Y_{1}\right]+\left[Y_{1}^{*}\right]+\left[C_{1}\right]+\left[C_{1}^{\prime }\right]+\left[C_{2}\right]+\left[C_{3}\right]$$

$$Y_{2T}=\left[Y_{2}\right]+\left[Y_{2}^{*}\right]+\left[C_{2}\right]+\left[C_{2}^{\prime }\right]$$

$$Y_{3T}=\left[Y_{3}\right]+\left[Y_{3}^{*}\right]+\left[C_{3}\right]+\left[C_{3}^{\prime }\right]+\left[C_{D}\right]$$

$$E_{k_{1T}}=\left[E_{k_{1}}\right]+\left[C_{1}\right]$$

$$E_{p_{1T}}=\left[E_{p_{1}}\right]+\left[C_{1}^{\prime }\right]$$

$$E_{p_{2T}}=\left[E_{p_{2}}\right]+\left[C_{2}^{\prime }\right]$$

$$E_{p_{3T}}=\left[E_{p_{3}}\right]+\left[C_{3}^{\prime }\right]$$

$$D_{T}=\left[D\right]+\left[C_{D}\right]$$

Dimensionless Parameters

$$P_{i}=\frac{k_{i}E_{k_{iT}}}{k_{i}^{\prime }E_{p_{iT}}}=\frac{V_{max_{k_{i}}}}{V_{max_{p_{i}}}}$$

$$E_{i}=\frac{E_{k_{iT}}}{Y_{iT}}\;{E^{\prime }}_{i}=\frac{E_{p_{iT}}}{Y_{iT}}$$

$$K_{i}=\frac{d_{i}+k_{i}}{a_{i}Y_{iT}}=\frac{K_{m_{k_{i}}}}{Y_{iT}}$$

$$K_{i}^{\prime }=\frac{{d^{\prime }}_{i}+{k^{\prime }}_{i}}{{a^{\prime }}_{i}Y_{iT}}=\frac{K_{m_{p_{i}}}}{Y_{iT}}$$

$$K_{B}=\frac{k_{off}∕k_{on}}{Y_{3T}}\left(\mathrm{for}\;n=3\right)$$

$$K_{B}=\frac{k_{off}∕k_{on}}{Y_{nT}}\left(\mathrm{for}\;\mathrm{all}\;n\right)$$

$$I=\frac{D_{T}}{Y_{3T}}\left(\mathrm{for}\;n=3\right)\;I=\frac{D_{T}}{Y_{nT}}\left(\mathrm{for}\;\mathrm{all}\;n\right)$$

$$\mathrm{where}\;E_{k_{i}T}=\left\{\begin{array}{c} i=1,\\ i=2,\;i=3,\\ i>3\;\left(\text{vertical}\right),\\ i>3\;\left(\text{lateral}\right), \end{array}\;\begin{array}{c} E_{k_{1T}}\\ Y_{1T}\\ Y_{\left(i-1\right)T}\\ Y_{1T} \end{array}\right)$$

Dimensionless Variables

$$y_{i}=\frac{\left[Y_{i}\right]}{Y_{iT}}\;y_{i}^{*}=\frac{\left[Y_{i}^{*}\right]}{Y_{iT}}$$

Algebraic rearrangement and substitution yield the following dimensionless steady state equations:

(1) $P_{1}\frac{y_{1}}{y_{1}+K_{1}}-\frac{y_{1}^{*}}{y_{1}^{*}+K_{1}^{\prime }}=0$

(2) $\begin{array}{c} -1+y_{1}+y_{1}^{*}\left(1+\frac{y_{2}}{K_{2}}+\frac{y_{3}}{K_{3}}\right)\\ \;+E_{1}\frac{y_{1}}{y_{1}+K_{1}}+E_{1}^{\prime }\frac{y_{1}^{*}}{y_{1}^{*}+K_{1}^{\prime }}=0 \end{array}$

(3) $P_{2}\frac{y_{2}y_{1}^{*}}{K_{2}}-\frac{y_{2}^{*}}{y_{2}^{*}+K_{2}^{\prime }}=0$

(4) $\begin{array}{c} -1+y_{2}\left(1+E_{2}\frac{y_{1}^{*}}{K_{2}}\right)+y_{2}^{*}\\ \;+E_{2}^{\prime }\frac{y_{2}^{*}}{y_{2}^{*}+K_{2}^{\prime }}=0 \end{array}$

(5) $P_{3}\frac{y_{3}y_{1}^{*}}{K_{3}}-\frac{y_{3}^{*}}{y_{3}^{*}+K_{3}^{\prime }}=0$

(6) $\begin{array}{c} -1+y_{3}\left(1+E_{3}\frac{y_{1}^{*}}{K_{3}}\right)+y_{3}^{*}\\ \;+E_{3}^{\prime }\frac{y_{3}^{*}}{y_{3}^{*}+K_{3}^{\prime }}+I\frac{y_{3}^{*}}{y_{3}^{*}+K_{B}}=0 \end{array}$

## Supplementary Material

Additional file 1 **Mapping dimensionless parameters to dimensional parameters**. This file describes how randomly sampled dimensionless parameter values were mapped to dimensional parameter values prior to numeric simulation. Click here for file

Additional file 2 **Parameter space sampling to estimate the probability of off-target effects**. This file describes how the parameter space of a network was sampled to provide an estimate of the probability of off-target effects due to retroactivity alone. Click here for file

Additional file 3 **Additional analysis of the *n *= 3 and *extended n *= 3 networks**. This file provides additional results from the numeric perturbation analyses of the *n *= 3 and *extended n *= 3 networks. Click here for file

Additional file 4 ***Xenopus *MAPK Model Parameters **[18,27,28]. This file explains how the *Xenopus *model parameters listed in Table 2 were derived. Click here for file
