# Supplementary material for: Kinase inhibitors can produce off-target effects and activate linked pathways by retroactivity
Source: BMC Syst Biol. 2011 Oct 4;5:156. doi: 10.1186/1752-0509-5-156 (PMC3257213; doi:10.1186/1752-0509-5-156)
Supplement: Additional file 1 — Mapping dimensionless parameters to dimensional parameters. This file describes how randomly sampled dimensionless parameter values were mapped to dimensional parameter values prior to numeric simulation. [file 1752-0509-5-156-S1.PDF]

Additional File 1 – Mapping dimensionless parameters to dimensional parameters

It was numerically more efficient (and in some cases more accurate) to solve the system of ODEs for a long period to ensure steady state was obtained rather than directly solving the set of non-linear steady state equations for each network.

Prior to solving the ODE system, a set of dimensionless parameter values was mapped to dimensional parameter values and initial conditions. The example below applies to the n = 3 network (all dimensionless parameters are in bold).

Initial conditions

$[Y_1] = 1 \qquad Y_{1T} = [Y_1] \qquad [Y_1^*] = 0$

$[Y_2] = Y_{1T}/\boldsymbol{E_2} \qquad Y_{2T} = [Y_2] \qquad [Y_2^*] = 0$

$[Y_3] = Y_{1T}/\boldsymbol{E_3} \qquad Y_{3T} = [Y_3] \qquad [Y_3^*] = 0$

$E_{k_{1T}} = \boldsymbol{E_1}\, Y_{1T} \qquad E_{p_{1T}} = \boldsymbol{E'_1}\, Y_{1T}$

$E_{p_{2T}} = \boldsymbol{E'_2}\, Y_{2T}$

$E_{p_{3T}} = \boldsymbol{E'_3}\, Y_{3T}$

$[C_1] = [C'_1] = [C_2] = [C'_2] = [C_3] = [C'_3] = [C_D] = 0$

Kinetic parameters

$k'_1 = k'_2 = k'_3 = 1$

$k_1 = (\boldsymbol{P_1}\, E_{p_{1T}}\, k'_1)/E_{k_{1T}}$

$k_2 = (\boldsymbol{P_2}\, E_{p_{2T}}\, k'_2)/Y_{1T}$

$k_3 = (\boldsymbol{P_3}\, E_{p_{3T}}\, k'_3)/Y_{1T}$

$d_1 = d'_1 = d_2 = d'_2 = d_3 = d'_3 = k_{on} = 1$

$a_1 = (d_1 + k_1)/(\boldsymbol{K_1}\, Y_{1T})$ $a'_1 = (d'_1 + k'_1)/(\boldsymbol{K'_1}\, Y_{1T})$

$a_2 = (d_2 + k_2)/(\boldsymbol{K_2}\, Y_{2T})$ $a'_2 = (d'_2 + k'_2)/(\boldsymbol{K'_2}\, Y_{2T})$

$a_3 = (d_3 + k_3)/(\boldsymbol{K_3}\, Y_{3T})$ $a'_3 = (d'_3 + k'_3)/(\boldsymbol{K'_3}\, Y_{3T})$

$k_{off} = \boldsymbol{K_B}\, Y_{3T}\, k_{on}$
