# Supplementary material for: Kinase inhibitors can produce off-target effects and activate linked pathways by retroactivity
Source: BMC Syst Biol. 2011 Oct 4;5:156. doi: 10.1186/1752-0509-5-156 (PMC3257213; doi:10.1186/1752-0509-5-156)
Supplement: Additional file 2 — Parameter space sampling to estimate the probability of off-target effects. This file describes how the parameter space of a network was sampled to provide an estimate of the probability of off-target effects due to retroactivity alone. [file 1752-0509-5-156-S2.PDF]

Additional File 2 – Parameter space sampling to estimate the probability of off-target effects

As described in the Methods section and in Additional File 2, Figure S1, 5,000 randomly selected parameter sets were used to determine a probability of off-target effects in a given parameter space. Random sampling was performed by latin hypercube sampling (LHS). When the default parameter ranges defined in Table 1 were used, the probability of off-target effects (i.e., the percentage of off-target effects in the randomly selected parameter sets) was 1.6%.

We also selected 50,000 parameter sets using the same ranges and found a probability of 1.5%. Histograms of all parameter values in the 50,000 sampled parameter sets indicate a uniform distribution across all ranges sampled as expected from the LHS method used (Additional File 2, Figure S2A). Histograms of the 745 parameter sets (out of 50,000) that produced off-target effects reveal specific ranges that favor an off-target effect in cycle 2 (Additional File 2, Figure S2B). These ranges are in general agreement with the ranges identified by the perturbation method in Figure 3.

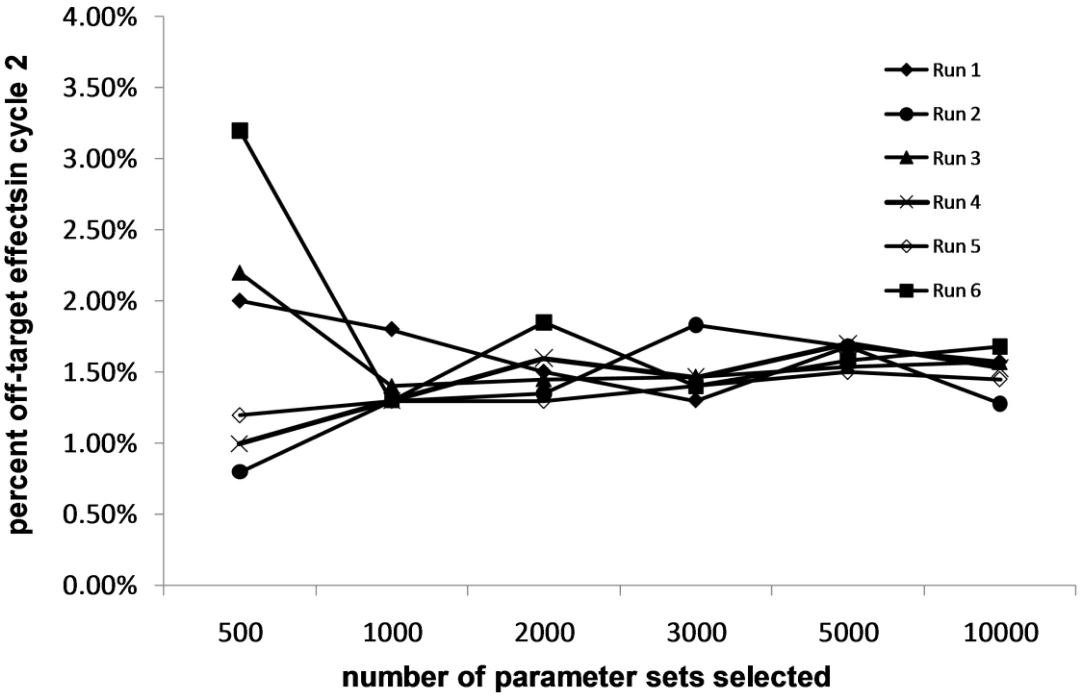

Figure S1 - Determination of the number of parameter sets to sample. The percentage of off-targets in the n = 3 network (Figure 2C) was measured in q randomly selected parameter sets using the full parameter ranges defined in Table 1. The values of q tested were 500, 1000, 2000, 3000, 5000, and 10,000. The test was repeated six times. The variation in the percentage off-target effects in the parameter space appeared to stabilize for repeats greater than or equal to 5000. We, therefore, selected 5000 as the number of parameter sets to sample. A similar test was performed with the *extended n = 3* network (Figure 2D). No substantial differences was found with the *extended n = 3* network when values of q tested were 5000 and 50,000 (data not shown).

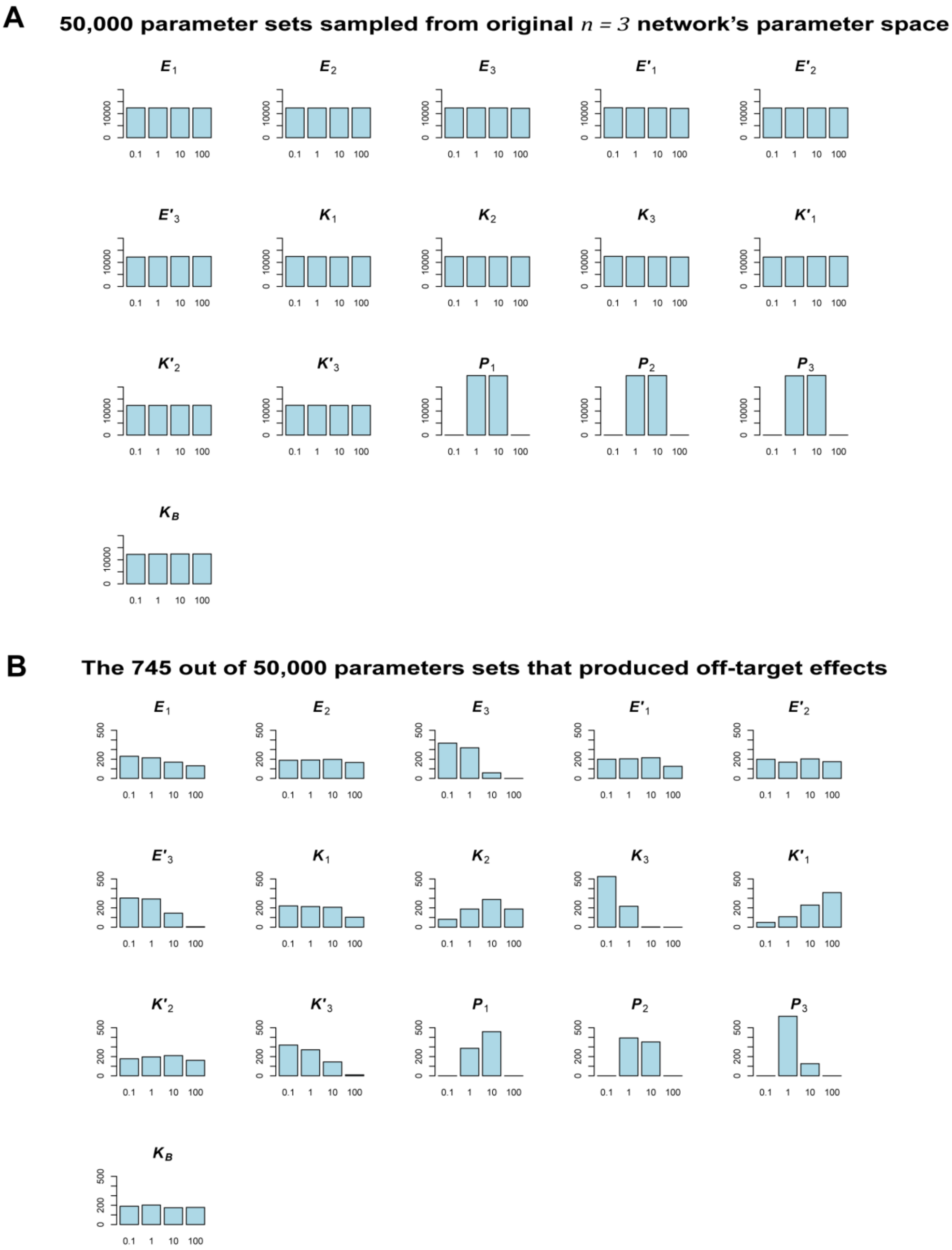

Figure S2 - Histograms of parameter values in 50,000 randomly sampled parameter sets in the n = 3 network. When 50,000 parameter sets were sample from the original $n = 3$ parameter space (depicted in Figure 3H and defined in Table 1). 1.5% of the sampled parameter sets produced off-target effects in cycle 2. This value is effectively the same as was found when we sampled 5,000 parameter sets. **(A)** Histograms of all parameter values in the 50,000 parameter sets sampled indicate uniform sampling distributions. **(B)** Histograms of the 745 out of the 50,000 parameter sets sampled which produced off-target effects suggest parameter ranges that favor an off-target effect in cycle 2 (compare to Figure 3).
