# Supplementary material for: Kinase inhibitors can produce off-target effects and activate linked pathways by retroactivity
Source: BMC Syst Biol. 2011 Oct 4;5:156. doi: 10.1186/1752-0509-5-156 (PMC3257213; doi:10.1186/1752-0509-5-156)
Supplement: Additional file 3 — Additional analysis of the n = 3 and extended n = 3 networks. This file provides additional results from the numeric perturbation analyses of the n = 3 and extended n = 3 networks. [file 1752-0509-5-156-S3.PDF]

Additional File 3 – Additional analysis of the *n* = 3 and *extended n* = 3 networks

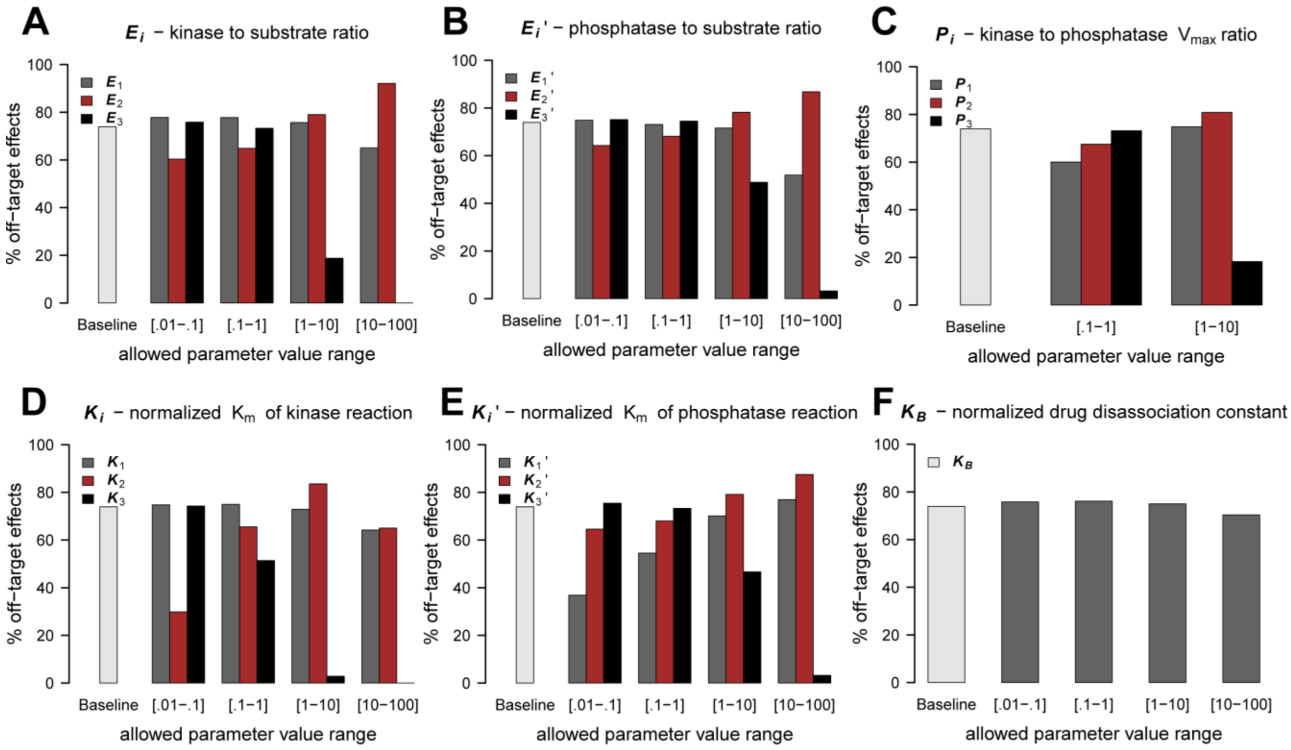

Figure S3 - A numeric perturbation analysis revealed parameter value ranges that promote off-target effects when the n = 3 network's parameter space was restricted. A perturbation analysis of the restricted parameter space (depicted in Figure 3I) of the n = 3 network was performed. The baseline in each plot is 74% (the percentage of off-target effects when 5000 parameter sets were randomly sampled from this parameter space). All other bars reflect the results of systematically perturbing each parameter (one at a time) using the given sub-ranges **(A-F)**.

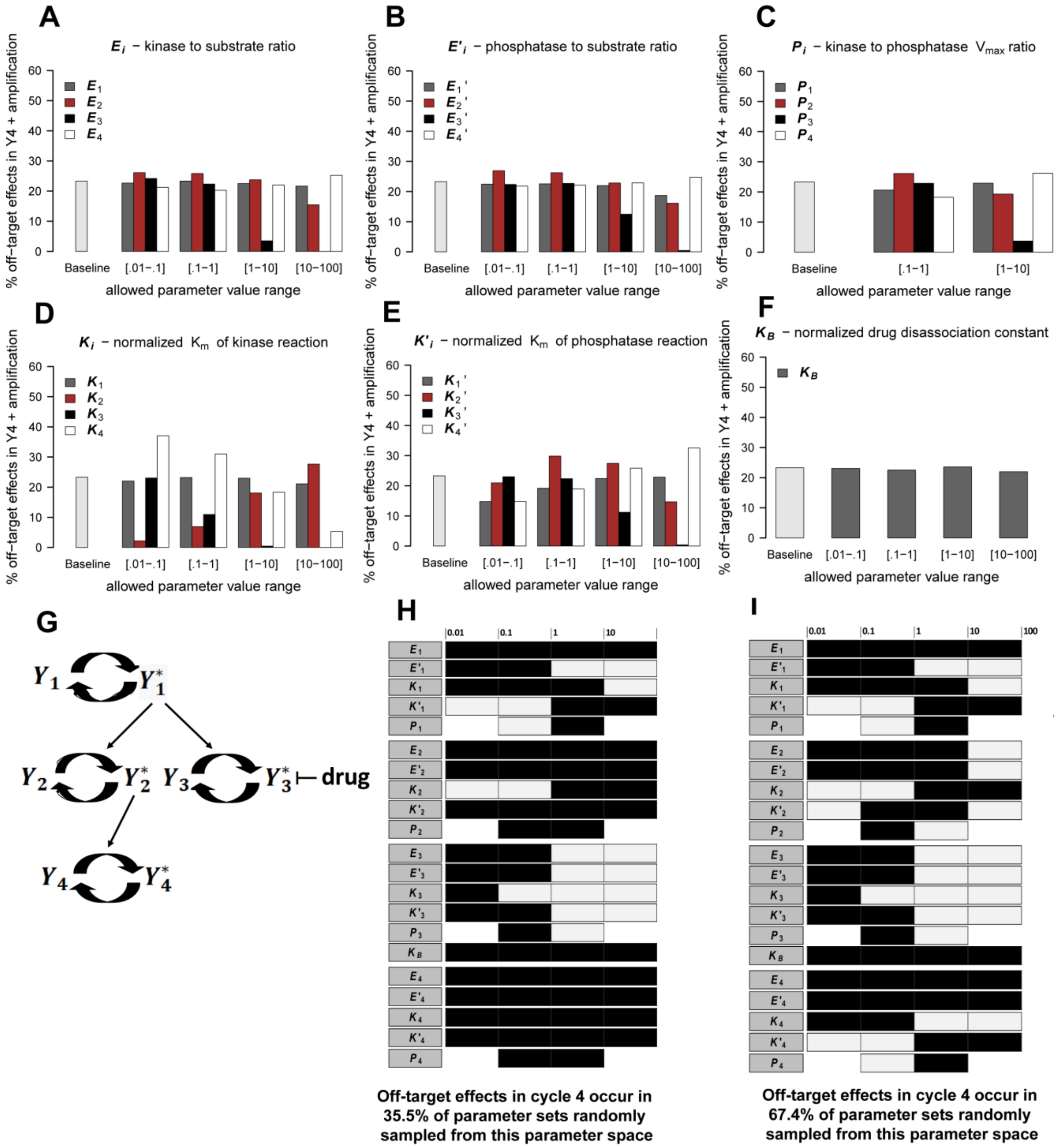

Figure S4 - A numeric perturbation analysis revealed parameter value ranges that promote off-target effects with amplification in *extended* *n* = 3 network. A perturbation analysis of the partially restricted parameter space (depicted in **H**) of the *extended* $n = 3$ network (**G**) was performed. The baseline in each plot is 23.4% (percentage of off-target effects amplified from cycle 2 to cycle 4 when 5000 parameter sets were randomly sampled). All other bars reflect the results of systematically perturbing each parameter (one at a time) using the given sub-ranges **(A-F)**. Finally, cycle 2 and cycle 4 parameter ranges were further restricted based on the results in **(A-F)** to produce a new parameter space **(I)** This new parameter space produced off-target effects in 67.4% of sampled parameter sets and produced off-target effects with amplification from cycle 2 to cycle 4 in 61.9% of sample parameter sets.

A numeric perturbation analysis (as described in the Methods) was performed on the *extended* $n = 3$ network parameter space depicted in Additional File 3, Figure S4H. In this analysis, off-target effects in cycle 4 which were amplified from cycle 2 were considered. The results indicated that a generally inefficient kinase reaction in cycle 2 ($\boldsymbol{K_2} >> 1$) favored amplification of off-target effects in cycle 4 (Additional File 3, Figure S4D). This result is similar to the behavior of $\boldsymbol{K_2}$ in the restricted $n = 3$ parameter space (Additional File 3, Figure S3D). In contrast, a more efficient kinase reaction in cycle 4 that operated near the zero-order regime ($\boldsymbol{K_4} < 1$) favored amplification of off-targets in cycle 4 (Additional File 3, Figure S4D). Amplification of off-target effects in cycle 4 were more likely for smaller values of $\boldsymbol{E_2}$ or $\boldsymbol{E'_2}$ (Additional File 3, Figure S4A-B). Because $\boldsymbol{E_2}$ is the total enzyme to substrate ratio of the kinase reaction in cycle 2 (i.e., Y_{1T}/Y_{2T}), these results suggest that amplification of off-target effects are more likely to propagate downstream from cycle 2 if the Y_2 protein pool is larger than the Y_1 protein pool. In contrast, off-target effects in cycle 2 were favored in the $n = 3$ network when $\boldsymbol{E_2}$ or $\boldsymbol{E'_2}$ was greater than 1 (i.e., the Y_2 protein pool was smaller than the Y_1 protein pool) (Additional File 3, Figure S3A-B).

The results also indicate that in the *extended* $n = 3$ network, analogous parameters in cycle 2 and cycle 4 work against each other. For example, large $\boldsymbol{K_2}$ and small $\boldsymbol{K_4}$ values contributed to amplification of off-target effects in cycle 4 (Additional File 3, Figure S4E). Similarly, large $\boldsymbol{P_4}$ and small $\boldsymbol{P_2}$ values tended to favor amplification of off-target effects in cycle 4 (Additional File 3, Figure S4C).
