# Supplementary material for: Kinase inhibitors can produce off-target effects and activate linked pathways by retroactivity
Source: BMC Syst Biol. 2011 Oct 4;5:156. doi: 10.1186/1752-0509-5-156 (PMC3257213; doi:10.1186/1752-0509-5-156)
Supplement: Additional file 4 — Xenopus MAPK Model Parameters [18,27,28]. This file explains how the Xenopus model parameters listed in Table 2 were derived. [file 1752-0509-5-156-S4.PDF]

Additional File 4 – *Xenopus* MAPK Model Parameters

The following values were extracted from the Huang and Ferrell [18] *Xenopus* model:

$E_{k_{1\mathrm{T}}} = 0.0003\mu\mathrm{M}$

$Y_{1\mathrm{T}} = 0.003\mu\mathrm{M}$ $Y_{2\mathrm{T}} = 1.2\mu\mathrm{M}$ $Y_{3\mathrm{T}} = 1.2\mu\mathrm{M}$

$E_{p_{1\mathrm{T}}} = 0.0003\mu\mathrm{M}$ $E_{p_{2\mathrm{T}}} = 0.0003\mu\mathrm{M}$ $E_{p_{3\mathrm{T}}} = 0.12\mu\mathrm{M}$

$\mathrm{K}_{\mathrm{m}_{k1}} = 0.3\mu\mathrm{M}$ $\mathrm{K}_{\mathrm{m}_{k2}} = 0.3\mu\mathrm{M}$ $\mathrm{K}_{\mathrm{m}_{k3}} = 0.3\mu\mathrm{M}$

$\mathrm{K}_{\mathrm{m}_{p_1}} = 0.3\mu\mathrm{M}$ $\mathrm{K}_{\mathrm{m}_{p_2}} = 0.3\mu\mathrm{M}$ $\mathrm{K}_{\mathrm{m}_{p_3}} = 0.3\mu\mathrm{M}$

$k_1 = 150\ \mathrm{min}^{-1}$ $k_2 = 150\ \mathrm{min}^{-1}$ $k_3 = 150\ \mathrm{min}^{-1}$

$k_1' = 150\ \mathrm{min}^{-1}$ $k_2' = 150\ \mathrm{min}^{-1}$ $k_3' = 150\ \mathrm{min}^{-1}$

$\mathrm{K_d}$ (the disassociation constant of the inhibitor binding to Y_3*) was not part of the original model and was arbitrarily set to .10 μM.

Substituting these values into the dimensionless parameter formulas found in Appendix A gives the values listed for the *Xenopus* parameter set in Table 2. The concentrations and first order rate constants used were based on published values in Huang and Ferrell [18], the representation of their MAPK model in the Biomodels repository [27] (http://www.ebi.ac.uk/biomodels-main/BIOMD0000000009), and a MAPK model published by Blüthgen and Herzel [28], which also used the Huang and Ferrell parameters. The Huang and Ferrell *Xenopus* model included double phosphorylation of the MAPK cascade, while our model only considered single phosphorylation, however.
